# Shroom3 functions downstream of planar cell polarity to regulate myosin II distribution and cellular organization during neural tube closure

**DOI:** 10.1242/bio.20149589

**Published:** 2015-01-14

**Authors:** Erica M. McGreevy, Deepthi Vijayraghavan, Lance A. Davidson, Jeffrey D. Hildebrand

**Affiliations:** 1 Department of Biological Sciences, University of Pittsburgh, Pittsburgh, PA 15260, USA; 2 Department of Bioengineering, University of Pittsburgh, Pittsburgh, PA 15260, USA

**Keywords:** Shroom3, Planar polarity, Neural tube, Myosin II, Apical constriction

## Abstract

Neural tube closure is a critical developmental event that relies on actomyosin contractility to facilitate specific processes such as apical constriction, tissue bending, and directional cell rearrangements. These complicated processes require the coordinated activities of Rho-Kinase (Rock), to regulate cytoskeletal dynamics and actomyosin contractility, and the Planar Cell Polarity (PCP) pathway, to direct the polarized cellular behaviors that drive convergent extension (CE) movements. Here we investigate the role of Shroom3 as a direct linker between PCP and actomyosin contractility during mouse neural tube morphogenesis. In embryos, simultaneous depletion of Shroom3 and the PCP components Vangl2 or Wnt5a results in an increased liability to NTDs and CE failure. We further show that these pathways intersect at Dishevelled, as Shroom3 and Dishevelled 2 co-distribute and form a physical complex in cells. We observed that multiple components of the Shroom3 pathway are planar polarized along mediolateral cell junctions in the neural plate of E8.5 embryos in a Shroom3 and PCP-dependent manner. Finally, we demonstrate that *Shroom3* mutant embryos exhibit defects in planar cell arrangement during neural tube closure, suggesting a role for Shroom3 activity in CE. These findings support a model in which the Shroom3 and PCP pathways interact to control CE and polarized bending of the neural plate and provide a clear illustration of the complex genetic basis of NTDs.

## INTRODUCTION

Formation of the central nervous system is critical for vertebrate embryogenesis. The brain and spinal cord develop from the neural tube, which forms through a series of morphogenetic events that transform the flat neural plate into a closed, hollow tube (Schoenwolf, 1991). Errors in this developmental program result in neural tube defects (NTDs), a class of highly prevalent congenital malformations. NTDs are classified based on the region of the neural tube affected and include spina bifida (posterior), exencephaly (cranial), and craniorachischisis (trunk) (Greene and Copp, 2009). There is evidence for a genetic basis of inheritance of NTDs, however, there are no known mutations in single genes that predispose a person to a NTD. Instead, their etiology is thought to result from a combination of risk alleles and environmental factors (Detrait et al., 2005). Despite their clinical importance, their causes are poorly understood.

During neural tube morphogenesis, cells within the neural plate intercalate towards the midline, extending the tissue along the anterior-posterior (AP) axis in a process known as convergent extension (CE) (Schoenwolf and Alvarez, 1989; Keller et al., 1992; Keller, 2002). CE requires cells to be polarized within the plane of the epithelium, a phenomenon known as Planar Cell Polarity (PCP). PCP was first described in *Drosophila*, in which PCP mutants exhibit disorganization of normally highly ordered epithelial structures (Gubb and García-Bellido, 1982; Wong and Adler, 1993). Disruption of PCP in vertebrates results in failed CE, giving rise to embryos with shortened AP axes and NTDs (Wallingford and Harland, 2002; Ciruna et al., 2006; Ybot-Gonzalez et al., 2007). PCP mutations in vertebrates also perturb ordered epithelial structures such as hair cells of the inner ear (Dabdoub and Kelley, 2005; Davies et al., 2005).

The propagation of PCP is controlled by a set of conserved proteins including the transmembrane proteins Frizzled (Fzd), and Van Gogh (Vang), the atypical cadherin Celsr (Flamingo in flies), and the cytoplasmic proteins Dishevelled (Dvl), Prickle (Pk), and Inversin (Diego in flies) (Seifert and Mlodzik, 2007; Zallen, 2007; Goodrich and Strutt, 2011; Gray et al., 2011). In *Drosophila*, PCP relies on the formation of membrane-localized protein complexes arranged asymmetrically at proximal and distal adherens junctions (Axelrod, 2001; Strutt, 2001; Bastock et al., 2003; Das et al., 2004; Chen et al., 2008). Asymmetric protein localization ensures that signaling is restricted to one pole of each cell, establishing polarity within the epithelium. Signaling proceeds via Dvl, which activates the small GTPase Rho to elicit polarized cytoskeletal rearrangements through the activation of Rho-Kinase (Rock) and non-muscle-Myosin II (Myosin II) (Habas et al., 2001; Winter et al., 2001; Marlow et al., 2002). The PCP pathway is initiated by Wnt ligands, which bind to Fzd receptors and transduce the signal to Dvl (Heisenberg et al., 2000; Tada and Smith, 2000; Kilian et al., 2003; Qian et al., 2007; Wu et al., 2013). PCP-dependent CE is critical for neural tube closure, as loss of function alleles of *Vangl2*, *Dvl* and *Dvl2*, *Celsr1*, and *Fzd3 and Fzd6* cause craniorachischisis (Greene et al., 1998; Curtin et al., 2003; Wang, Y. et al., 2006; Etheridge et al., 2008). Until recently, evidence for asymmetric localization of PCP components in the neural plate was lacking. Studies in chicken embryos have identified planar polarized Celsr1 and Dvl2, which localize to adherens junctions oriented along the mediolateral axis of the embryo (Nishimura and Takeichi, 2008; Nishimura et al., 2012). This results in planar polarization of Rock and phosphorylated myosin regulatory light chain (pMRLC). Cell intercalation during CE of the neural plate is thought to occur through a rosette-based mechanism, in which cells align locally to form multicellular units that form and resolve in a directed fashion (Williams et al., 2014). Accumulation of Rock and pMRLC at mediolateral cell junctions drives the formation of rosettes along this axis to promote directed cell intercalation and subsequent CE of the tissue.

In conjunction with CE, the neural plate also undergoes folding. In the neuroepithelium, prior to closure, the actin-binding protein Shroom3 colocalizes with F-actin at the zonula adherens (Hildebrand and Soriano, 1999; Haigo et al., 2003; Hildebrand, 2005). Through direct interaction, Shroom3 binds and recruits Rock, which activates Myosin II (Mohan et al., 2012; Mohan et al., 2013). Activation of Myosin II results in contraction of the F-actin network and subsequent apical constriction, a cell shape change that guides neural plate bending (Haigo et al., 2003; Hildebrand, 2005). Neural plate bending only occurs along the mediolateral axes of the embryo, indicating that the molecular machinery that drives plate bending is likely to exhibit planar polarity. Inhibition of Shroom3-Rock interactions within the neural plate of chicken embryos disrupts planar cell arrangement, including the formation of rosettes, indicating that Shroom3 may participate in both CE and apical constriction (Nishimura and Takeichi, 2008).

We employed a genetic approach in mice to address the hypothesis that Shroom3 and PCP function together to coordinate CE with polarized bending of the neural plate. *Shroom3* interacts genetically with the PCP components *Vangl2* and *Wnt5a* and these pathways appear to be linked by a complex of Dvl2, Shroom3, and Rock. We provide evidence that this complex is planar polarized along the mediolateral axis of the neuroepithelium and may regulate cellular organization and tissue bending to promote neural tube closure.

## RESULTS

### *Shroom3* interacts genetically with *Vangl2*

**Fig. 1.**
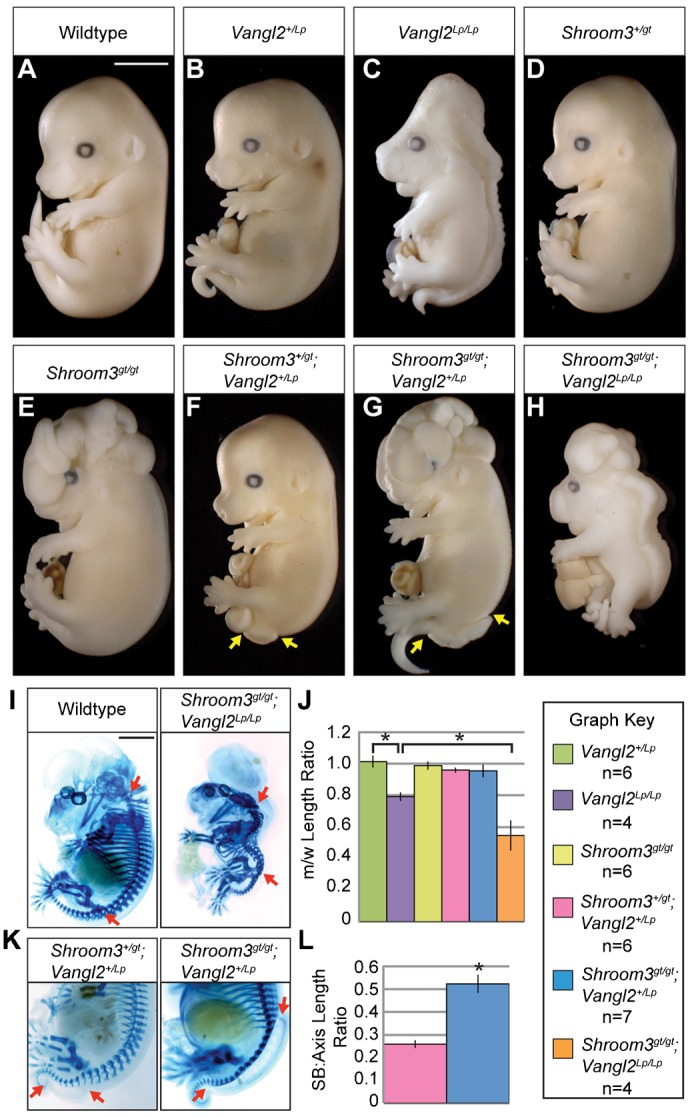
Shroom3 interacts genetically with *Looptail*. (A–H) Embryos of the indicated genotypes were isolated at E13.5 and observed in whole mount. NTD penetrance calculations were determined using a minimum n = 4. Yellow arrows denote spina bifida. (I) Axis lengths were measured from the 2^nd^ cervical vertebrae to the pelvic girdle (between red arrows) and normalized to a wildtype littermate. (J) Quantification of axis length, expressed as a mutant:wildtype ratio, m/w. (K) Typical spina bifida (between red arrows) phenotypes seen in *Shroom3^+/gt^;Vangl2^+/Lp^* and *Shroom3^gt/gt^;Vangl2^+/Lp^* embryos. (L) Quantification of spina bifida severity, expressed as the ratio of the length of the open spinal cord to the length of the body axis. Scale bars: 1 mm. *p<0 .001; Error bars ± s.e.m.

To identify novel genetic interactions between Shroom3 and PCP, we took advantage of a highly studied mouse model of NTDs, the *Looptail* (*Lp*) strain, which harbors a mutation in the core PCP factor *Vangl2* (Kibar et al., 2001). The *Lp* allele acts in a gene-dosage dependent manner such that heterozygous animals (*Vangl2^+/Lp^*) are viable but exhibit a looped tail, while homozygous embryos (*Vangl2^Lp/Lp^, Lp* mutant) die *in utero* due to craniorachischisis (Fig. 1A–C) (Smith and Stein, 1962). Embryos heterozygous for the *Shroom3* null allele (*Shroom3^+/gt^*) are normal, whereas homozygous embryos (*Shroom3^gt/gt^*, *Shroom3* mutant) exhibit exencephaly (Fig. 1D,E) (Hildebrand and Soriano, 1999). *Shroom3^+/gt^;Vangl2^+/Lp^* embryos develop spina bifida with a penetrance of 37.5% (9 out of 24) (Fig. 1F). *Shroom3^gt/gt^;Vangl2^+/Lp^* embryos exhibit exencephaly and severe spina bifida that drastically increases in penetrance to 75% (9 out of 12) (Fig. 1G). *Shroom3^gt/gt^*;*Vangl2^Lp/Lp^* embryos display an open neural tube and a distinct shortening of the AP axis (Fig. 1H).

The decreased AP length observed in PCP mutants is attributed to defective CE (Wang, J. et al., 2006; Ybot-Gonzalez et al., 2007; Mahaffey et al., 2013). Therefore, axis length measurements were used to determine whether disruption of *Shroom3* and PCP signaling induces stronger CE phenotypes than depletion of either independently. We measured the length of the neural tube from the 2^nd^ cervical vertebrae to the pelvic girdle (Fig. 1I, red arrows) and the axis length of mutants was normalized to that of a wildtype littermate to determine the mutant:wildtype (m/w) ratio. Our data demonstrate that *Lp* mutants exhibit a significant decrease in axis length compared to wildtype embryos and *Shroom3/Lp* double mutants exhibit a further decrease compared to *Lp* mutants (Fig. 1J). These data indicate that loss of Shroom3 function augments the CE defect caused by the loss of PCP signaling.

In addition to reduced axis length, we also observe increased severity of spina bifida upon increasing genetic insult. To quantify this phenotype, we measured the length of the spina bifida in *Shroom3^+/gt^;Vangl2^+/Lp^* or *Shroom3^gt/gt^;Vangl2^+/Lp^* embryos and these were normalized to the AP axis length of each embryo (Fig. 1K, red arrows). *Shroom3^gt/gt^;Vangl2^+/Lp^* embryos exhibit a significant increase in the length of the open neural tube as compared to *Shroom3^+/gt^;Vangl2^+/Lp^* embryos (Fig. 1L), suggesting that simultaneous disruption of Shroom3 and PCP increases both the frequency and severity of NTDs.

### *Shroom3* interacts genetically with *Wnt5a*

**Fig. 2.**
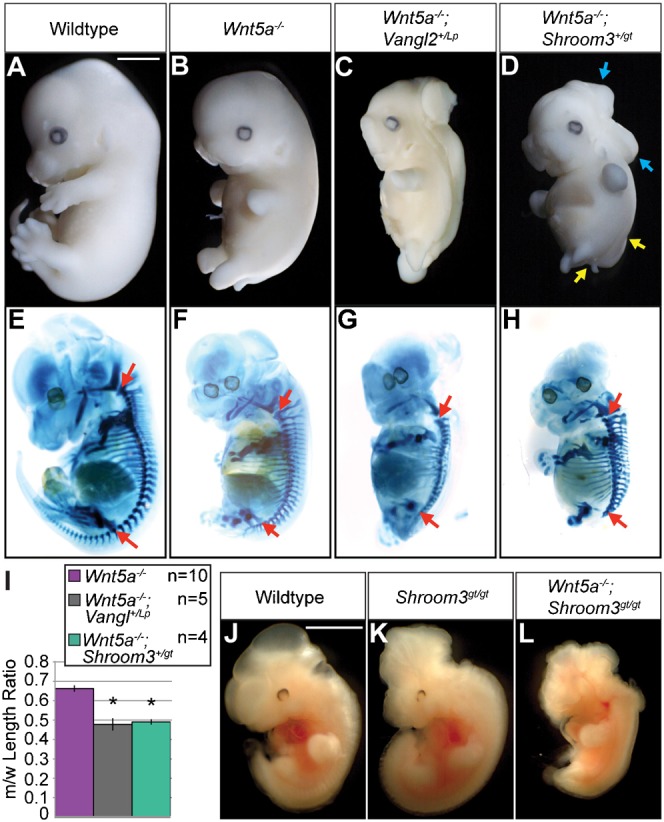
*Shroom3* interacts genetically with *Wnt5a*. (A–H) Embryos of the indicated genotypes were isolated at E12.5 and observed in whole mount (A–D) and processed to show the cartilaginous skeleton (E–H). NTD penetrance calculations were determined using a minimum n = 4. Blue and yellow arrows denote exencephaly and spina bifida, respectively. (I) AP axis lengths were measured from the 2^nd^ cervical vertebrae to the pelvic girdle (red arrows in panels E–H) and normalized to a wildtype littermate (m/w ratio). *p<0.001. Error bars: ±s.e.m. (J–L) Embryos of the indicated genotype were isolated at E11.5 and observed in whole mount. *Shroom3^gt/gt^;Wnt5a^−/−^:* n = 3. Scale bars: 1 mm.

To further establish the interaction between Shroom3 and PCP, we employed the same approach to assess the genetic interaction between *Shroom3* and *Wnt5a*. Wnt5a is a putative ligand for vertebrate PCP signaling and *Wnt5a* null mice exhibit characteristic CE phenotypes, although NTDs are only observed at a low frequency (Fig. 2A,B) (Qian et al., 2007). However, *Wnt5a^−/−^;Vangl2^+/Lp^* embryos exhibit craniorachischisis with a penetrance of 100%, implicating Wnt5a in PCP regulation (Fig. 2C) (Qian et al., 2007). *Wnt5a*^−/−^;*Shroom3^+/gt^* embryos develop exencephaly with a penetrance of 33% (3 out of 9, Fig. 2D, blue arrows) and spina bifida (Fig. 2D, yellow arrows). Axis length measurements revealed CE defects in *Wnt5a^−/−^;Vangl2^+/Lp^* and *Wnt5a^−/−^;Shroom3^+/gt^* embryos as compared to *Wnt5a^−/−^* (Fig. 2E–I). Dorsal views of skeletal preparations (not shown) demonstrate that all mutant and wildtype embryos have the same number of vertebrae between the first cervical vertebrae and the pelvic girdle, thus the decrease in axis length is due to defective axial extension, not truncation of the A-P axis due to errors in developmental processes such as somitogenesis. While difficult to measure accurately due to the severe excencephaly phenotype, it does not appear that there are axial elongation defects in the anterior portion of these embryos (data not shown). Since no double mutants were recovered at E13.5, we isolated embryos at E11.5 and recovered a small number of *Wnt5a^−/−^;Shroom3^gt/gt^* embryos that die at or before E11.5 with obvious NTDs, shortened body axes, and decreased outgrowth of numerous tissues (Fig. 2J–L). Although the cause of death is unknown, these phenotypes are more severe than what would be expected from either mutation alone.

### Dvl2 regulates the distribution of Shroom3 and Rock

Dvl proteins are key components of canonical and non-canonical Wnt signaling. Dvl proteins have three conserved domains; an N-terminal DIX domain, a central PDZ domain, and a C-terminal DEP domain (Wallingford and Habas, 2005). The DIX domain regulates the ability of Dvl to polymerize into protein assemblies, which correlates with the ability to stimulate canonical Wnt signaling through β-catenin dependent transcription (Schwarz-Romond et al., 2005; Schwarz-Romond et al., 2007). As a result of polymerization, both endogenous and ectopically expressed Dvl proteins have been observed in cytoplasmic puncta in cell culture and animal models including frogs, flies, mice, and sea urchins. These puncta can be recruited to the plasma membrane of cells through Fzd receptors. Live cell imaging and biochemical analysis of the DIX domain revealed that Dvl puncta are mobile and highly dynamic, rapidly assembling, growing, and disassembling over time, which can be attributed to the inherent ability of the DIX domain to polymerize in a reversible fashion. (Yanagawa et al., 1995; Miller et al., 1999; Torres and Nelson, 2000; Chang et al., 2005; Leonard and Ettensohn, 2007; Na et al., 2007; Matsumoto et al., 2010; Yu et al., 2010; Panousopoulou et al., 2013). The PDZ domain interacts with Fzd receptors to transduce Wnt signals while the DEP domain is essential for PCP signaling (Axelrod et al., 1998; Tada and Smith, 2000; Wallingford et al., 2000; Wong et al., 2003; Punchihewa et al., 2009; Simons et al., 2009). Dvl2 is sufficient for mouse neural tube closure, as *Dvl1/Dvl3* null embryos do not display NTDs while *Dvl1/Dvl2* or *Dvl2/Dvl3* null animals exhibit craniorachischisis (Wang, J. et al., 2006; Etheridge et al., 2008). Shroom3 is characterized by a specific linear array of motifs, including the N-terminal PDZ domain, the serine-proline rich region (SPR), the central actin-binding domain (Shroom-Domain 1, SD1), and the C-terminal Shroom-domain 2 (SD2) that directly binds to Rock. We have shown that the PDZ, SPR, and SD1 elements control subcellular localization while the SD2 regulates their *in vivo* activity (Hildebrand, 2005; Dietz et al., 2006).

**Fig. 3.**
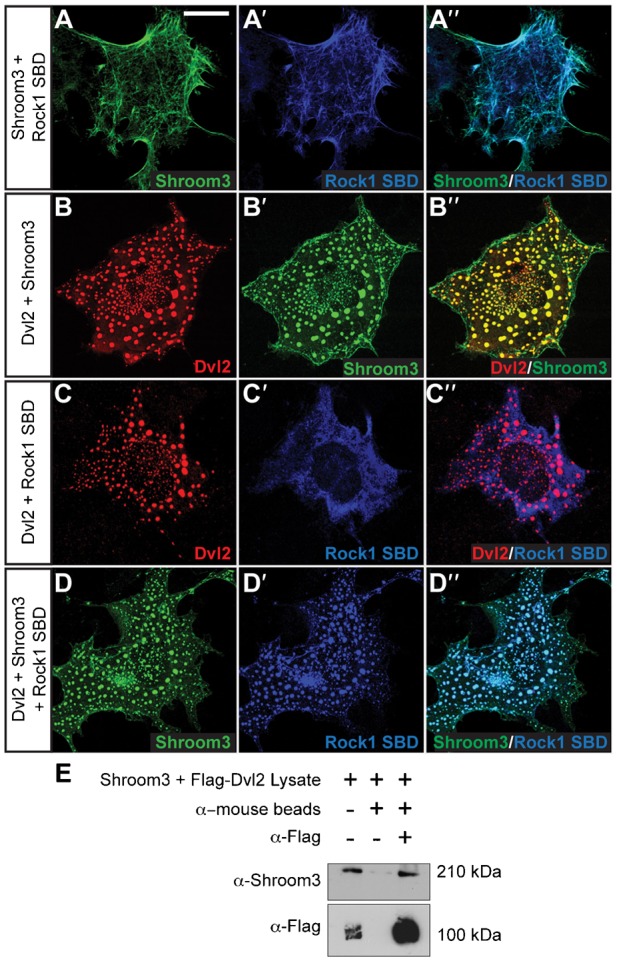
Dvl2 recruits Shroom3 and Rock into cytoplasmic puncta. (A–D) COS7 cells transiently expressing either Shroom3, Dvl2, and/or the Shroom Binding Domain (SBD) of Rock were grown on cover slips overnight and stained to detect the indicated proteins. Scale bar: 10 µm. Panels show representative images; qualitative results are based on three independent experiments. For panels A–D, individual channels are shown in the first two panels and the merge is shown in the last panel (A, Shroom3; A′, Rock1 SBD; A″, merge; B, Dvl2; B′, Shroom3; B″, merge; C, Dvl2; C′, Rock1 SBD; C″, merge; D, Shroom3; D′, Rock1 SBD, D″, merge). (E) Dvl2 was precipitated from lysates of COS7 cells co-expressing Shroom3 and Flag-Dvl2 with anti-flag antibodies followed by SDS–PAGE and immunoblotting to detect Shroom3 and Flag. MW markers are indicated to the right.

To investigate the connection between the Shroom3 and PCP pathways, we analyzed if their constituents co-localize in cells. When full length Shroom3 is expressed in COS7 cells, it binds actin filaments through a direct interaction with the SD1, resulting in co-localization with actin stress fibers (Fig. 3A) (Hildebrand, 2005). Consistent with previous results, co-expression of Shroom3 and the central coiled-coiled region of Rock results in the co-distribution of these proteins on actin stress fibers via the SD2 of Shroom3 and the Shroom-Binding Domain (SBD) of Rock (Fig. 3A) (Mohan et al., 2012; Mohan et al., 2013). When Dvl2 and Shroom3 are co-expressed, Shroom3 is recruited to Dvl2 puncta (Fig. 3B). In contrast, when Dvl2 is co-transfected with the Rock SBD, Rock is distributed in the cytoplasm (Fig. 3C). When Dvl2, Shroom3, and the Rock SBD are co-expressed, we observe a clear co-localization of Shroom3 and Rock in Dvl2 puncta (Fig. 3D). These data suggest that Dvl2 can regulate the localization of a Shroom3-Rock complex. To further verify this interaction and determine if Dvl2 and Shroom3 are in a complex, Dvl2 was immunoprecipitated from cells expressing both Dvl2 and Shroom3 and the immune complexes subjected to western blotting to detect Shroom3. This analysis shows a clear enrichment of Shroom3 in the immune complexes containing Dvl2 (Fig. 3E). In total, these data suggest that Dvl can direct the distribution of Shroom3 in cells and that a population of these proteins may exist in a common subcellular compartment or complex.

### The DEP domain and the N-terminal region of Shroom3 are required for complex formation

**Fig. 4.**
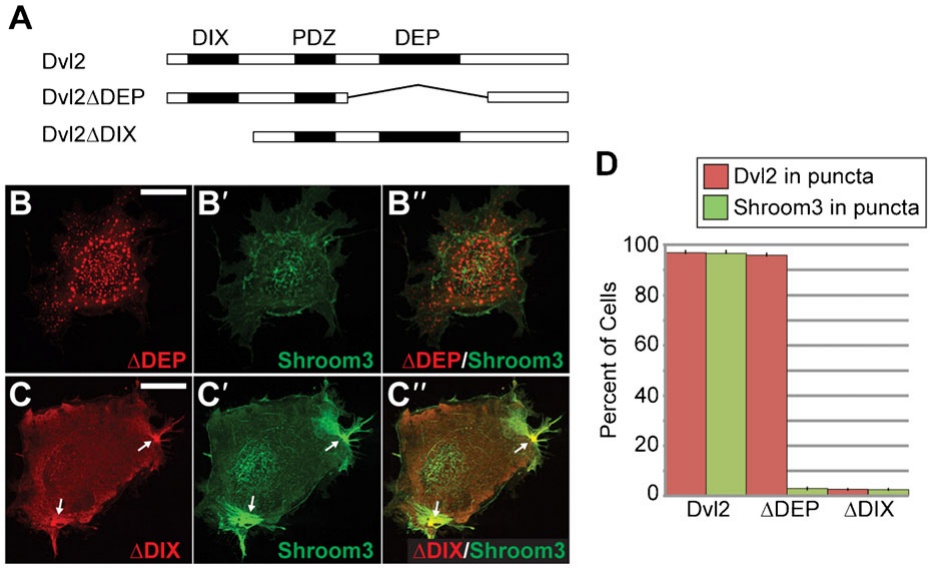
The DEP domain of Dvl2 facilitates interaction with Shroom3. (A–C) Mapping the Shroom3 binding region of Dvl2. Indicated deletion mutants of Dvl2 (A) and Shroom3 were transiently coexpressed in COS7 cells and detected via immunofluorescent microcopy (B,C). Individual channels are shown in the first two panels and the merge is shown in the last panel (B, Dvl2ΔDEP; B′, Shroom3; B″, merge; C, Dvl2ΔDIX; C′, Shroom3; C″, merge). Deletion of the DEP domain prevents recruitment of Shroom3 into puncta. Deletion of the DIX domain prevents Dvl2 polymerization and thus no puncta are observed. Some Dvl2/Shroom3 colocalization is observed at actin stress fibers (C, arrows) Scale bars: 10 µm. (D) Quantification of Shroom3 recruitment into puncta. Graph shows the percent of cells that show Dvl2 or Shoorm3 localized to puncta in 200 cells from three independent experiments. Error bars: ±s.e.m.

The above data suggest that Dvl2 may be the intersection between the Shroom3 and PCP pathways. To further characterize this interaction, we used deletion constructs to map the regions of Dvl2 and Shroom3 that mediate co-localization. Flag-tagged variants of Dvl2 lacking either the DEP or DIX domains were co-expressed with full length Shroom3 in COS7 cells and co-localization was evaluated by immunohistochemistry (Fig. 4). As expected, based on previous studies, the ΔDEP protein retains the ability to form cytoplasmic puncta while the ΔDIX protein no longer forms puncta and is largely cytoplasmic (Fig. 4B,C) (Schwarz-Romond et al., 2005). When assessed for Shroom3 distribution, we observe that the ΔDEP protein fails to recruit Shroom3 into these puncta (Fig. 4B). In contrast, in cells expressing the ΔDIX protein, Shroom3 is largely localized to actin-rich structures and appears to co-distribute with a population of Dvl2 (Fig. 4C, white arrows). To quantify this data, the percent of cells that show localization of Dvl2 and/or Shroom3 to cytoplasmic puncta was determined by counting 200 cells in three independent experiments. Deletion of the DEP domain prevents recruitment of Shroom3 into cytoplasmic puncta in nearly 100% of the cells (Fig. 4D). These data suggest that the DEP domain is required for the co-distribution of Shroom3 and Dvl2.

**Fig. 5.**
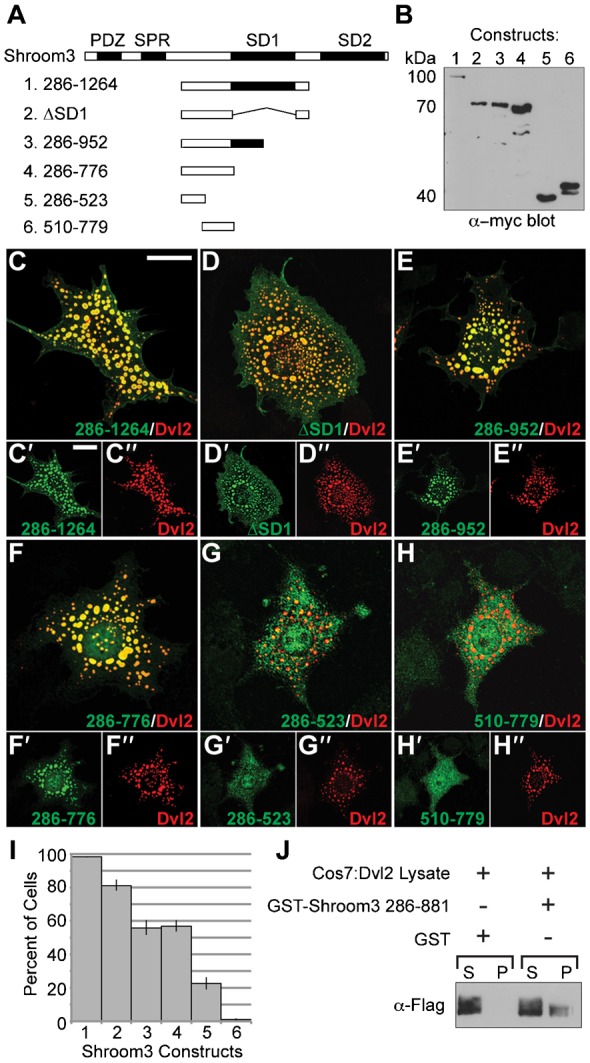
An uncharacterized region of Shroom3 mediates colocalization with Dvl2. (A–H) Dvl2 and the indicated myc-tagged Shroom3 proteins (A) were coexpressed in COS7 cells and detected via Western blotting (anti-Myc blot, B) or immunofluorescent microscopy and analyzed for colocalization in puncta. Merged images are shown in large panels, individual fluorescent channels are shown below the merged images (C–H, ′ and ″ denote individual fluorescent channels used to generate the merged image). Scale bars: 10 µm. (I) Quantification of Shroom3 recruitment into puncta. Graph shows the percent of cells that show Dvl2/Shroom3 colocalization in puncta in 200 cells from three independent experiments. Error bars: ±s.e.m. (J) GST or GST-Shroom3 286–881 bound to beads were added to COS7 lysate containing Dvl2, incubated, washed, pelleted, resolved by SDS-PAGE, and subjected to western blotting to detect Flag-tagged Dvl2. S, supernatant; P, pellet.

A series of deletion constructs were similarly employed to identify the region of Shroom3 that mediates co-localization with Dvl2 (Fig. 5A,B). Co-localization of Shroom3 and Dvl2 is maintained in the absence of the PDZ, SPR, SD1, and SD2 motifs, implicating an uncharacterized region spanning amino acids 286–776 in mediating the interaction between Shroom3 and Dvl2 (Fig. 5C–F). Constructs that disrupt this region abolish co-localization with Dvl2 (Fig. 5G,H). We quantified this data by counting the number of cells in which the indicated Shroom3 protein is recruited to Dvl2 puncta (Fig. 5I). To show that this region of Shroom3 can form a complex with Dvl2, we performed a GST pull-down assay using amino acids 286–881 of Shroom3 and cell lysates containing Dvl2 (Fig. 5J). Data from these experiments show this region of Shroom3 can form a complex containing Dvl2. These results indicate that Shroom3 and Dvl2 may interact to link PCP signaling to actomyosin contractility during neural tube morphogenesis.

### The Shroom3 pathway is enriched at mediolateral cell junctions

**Fig. 6.**
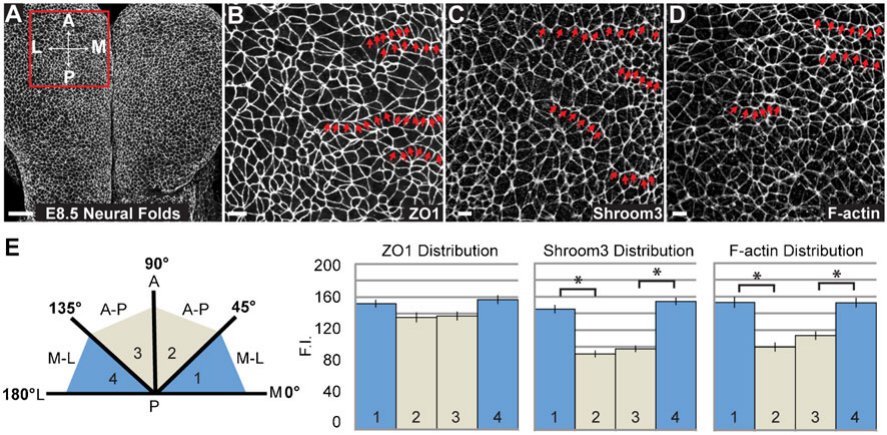
Shroom3 and F-actin are enriched at mediolateral junctions. (A) Schematic of analytical approach. Wildtype E8.5 embryos were stained in whole mount to detect ZO1 and visualize apical cell junctions of neuroepithelial cells. Scale bar: 50 µm. M = medial, L = lateral, A = anterior, P = posterior. Box indicates the region of the embryo used for quantification in panels B–E. (B–D) Embryos were stained to detect ZO1 (B) Shroom3 (C) or F-actin (D). Red arrows show examples of cells linked linearly along their mediolateral junctions. Scale bars: 10 µm. (E) Quantification of polarized protein distribution of ZO1, Shroom3, and F-actin. n≥200 junctions from at least three embryos; *p<0.001; Error bars: ±s.e.m.

**Fig. 7.**
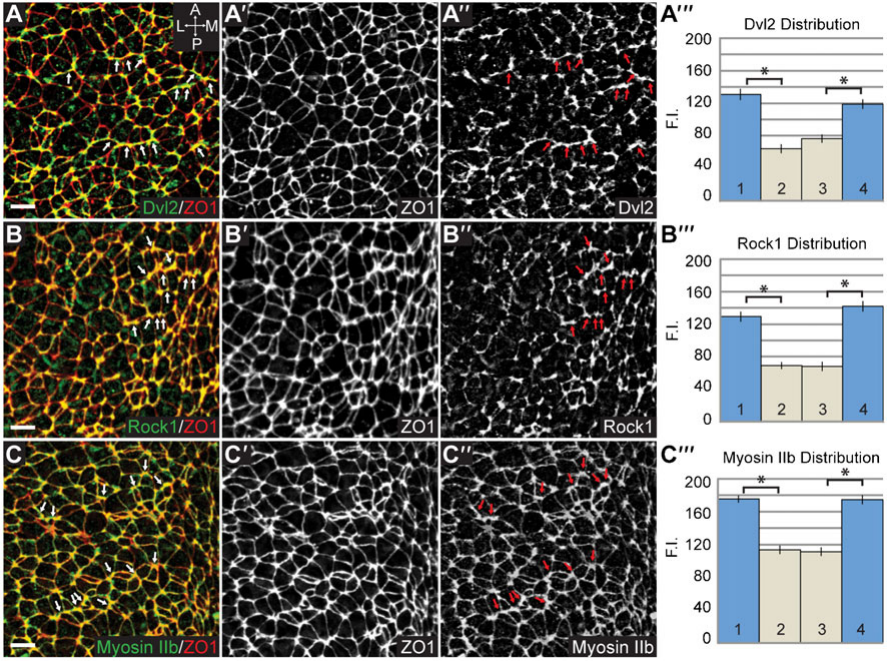
Myosin IIb, Rock1, and Dvl2 are planarly distributed in the neural plate. (A–C) Wildtype E8.5 embryos were co-stained to visualize ZO1 with Dvl2, Rock1, or Myosin IIb. Arrows show enrichment along M–L junctions; scale bars: 10 µm. For a panels A–C, ′ and ″ denote individual fluorescent channels used to generate the merged image. Graphs show quantification of planar distribution analysis. n≥200 junctions from at least three embryos, *p<0.001; Error bars: ±s.e.m.

We hypothesized that PCP and Shroom3 cooperate to regulate the distribution of contractile networks to promote cellular organization and directional bending of the neural plate. To investigate this, we stained neural plates from E8.5 wildtype mouse embryos to detect ZO1, Shroom3, and F-actin (Fig. 6A–D). While cells of the cranial neural plate are not highly organized, we do observe groups of neighboring cells linearly aligned to form contiguous cell junctions oriented along the mediolateral axis (Fig. 6B, red arrows). Importantly, both Shroom3 and F-actin are enriched along these junctions (Fig. 6C,D, red arrows). We quantified this distribution by measuring the fluorescence intensity of each cell-cell junction relative to the mediolateral or AP axes of the embryo as described in the material and methods (Fig. 6E). These data show a significant enrichment of Shroom3 and F-actin in junctions oriented along the mediolateral axis (Fig. 6E). In light of previous work showing the genetic and biochemical interactions between Shroom3, Dvl2, Rock, and Myosin II, we hypothesized that these factors would be similarly planarly distributed in the neuroepithelium. In wildtype E8.5 embryos, Dvl2, Rock1 and Myosin IIb are all enriched at mediolateral cell junctions (Fig. 7A–C).

### PCP is upstream of Shroom3

**Fig. 8.**
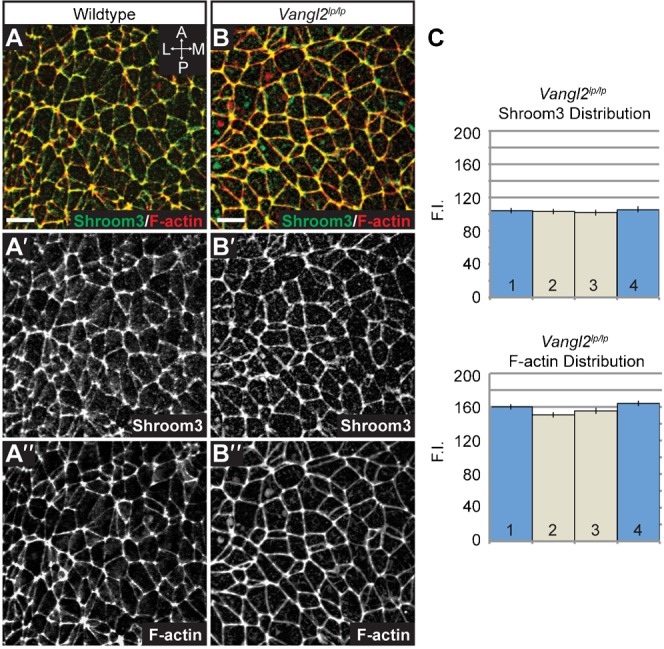
Planar distribution of F-actin and Shroom3 is lost in *Lp* mutants. (A,B) Wildtype and *Vangl2^Lp/Lp^* E8.5 embryos were co-stained in whole mount to visualize the distribution of Shroom3 and F-actin. For panels A and B, ′ and ″ denote individual fluorescent channels used to generate the merged image. Scale bars: 10 µm. (C) Quantification of planar distribution of Shroom3 and F-actin in *Lp* mutants. n≥200 junctions from at least three embryos/genotype.

**Fig. 9.**
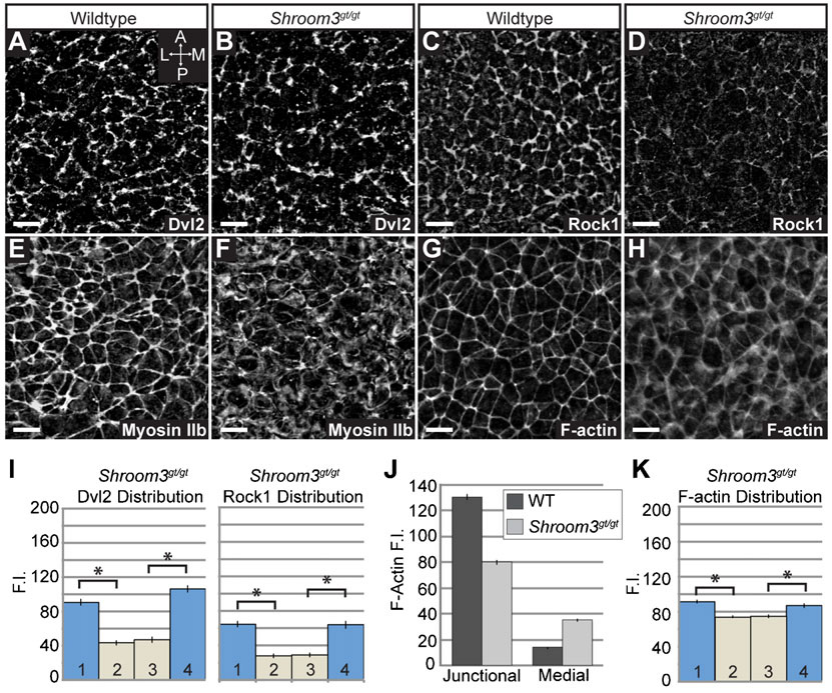
Analysis of Shroom3 and PCP pathway components in *Shroom3* mutant embryos. (A–H) Wildtype (A,C,E,G) and *Shroom3^gt/gt^* (B,D,F,H) neural plates were stained to visualize the planar distribution of Dvl2 (A,B), Rock1 (C,D), Myosin IIb (E,F) and F-actin (G,H). Scale bars: 10 µm. (I) Quantification of planar distribution of Dvl2 and Rock1. Dvl2 and Rock1 exhibit planar polarization, however a loss in total fluorescence intensity is apparent. n≥200 junctions from at least three embryos of each genotype. (J,K) Quantification of F-actin distribution in *Shroom3^gt/gt^* neural plates. User-drawn ROIs for the cell junctions of 300 cells in 3 wildtype and 3 *Shroom3^gt/gt^* embryos were used to determine the fluorescent intensity of F-actin at cell-cell junctions. Graph in (J) shows a loss of F-actin at apical junctions and an increase in medial actin populations in *Shroom3^gt/gt^* neural plates compared to wildtype. The remaining junctional F-actin is mediolaterally enriched in *Shroom3* mutants (K). In all cases, *p<0.001, Error bars: ±s.e.m.

To determine if Shroom3 is downstream of PCP signaling, we stained E8.5 *Shroom3^gt/gt^* and *Vangl2^Lp/Lp^* embryos to detect components of these pathways (Fig. 8A,B, Fig. 9A–H). In *Lp* mutants, Shroom3 and F-actin are localized to cell-cell junctions but are no longer enriched at mediolateral junctions, indicating that the general localization signals are intact, but mediolateral asymmetry of the adherens junctions is lost (Fig. 8A–C vs Fig. 6C–E). In *Shroom3^gt/gt^* embryos, Dvl2 is recruited to junctions and remains planarly polarized (Fig. 9A,B), although the amount of Dvl2 recruited to cell junctions may be reduced (Fig. 7A vs Fig. 9I). Rock1 localization is significantly impaired in *Shroom3^gt/gt^* embryos (Fig. 9C–F) but the remaining Rock1 is mediolaterally enriched, suggesting there are other mechanisms for localization (Fig. 9I). Consistent with the loss of Rock1 at cell junctions, we see reduced junctional Myosin IIb and increased cytoplasmic Myosin IIb (Fig. 9E,F). This increased cytoplasmic staining prevents accurate quantification of the Myosin IIb distribution. These results provide evidence that Shroom3 recruits Rock1 and Myosin IIb to adherens junctions while the PCP pathway refines the spatial distribution of the pathway.

Based on the above results, we assessed if Shroom3 loss affects the apical actin cytoskeleton of the neuroepithelium. Shroom3 deficiency results in a diffuse, discontinuous apicojunctional F-actin network and the appearance of medial or cytoplasmic F-actin (Fig. 9G,H). In wildtype embryos, 90% of the F-actin fluorescence is localized along cell-cell junctions. *Shroom3^gt/gt^* embryos exhibit an approximate 40% decrease in the fluorescence intensity of junctional F-actin and a concomitant increase in the medial population (Fig. 9J). Consistent with what we observe for Rock1, the remaining junctional F-actin exhibits a distribution bias along the mediolateral axis (Fig. 9K). These data demonstrate that Shroom3 functions downstream of PCP to regulate the distribution of Rock and Myosin II, which may feed back to regulate F-actin organization.

### Loss of Shroom3 disrupts planar cell arrangement

**Fig. 10.**
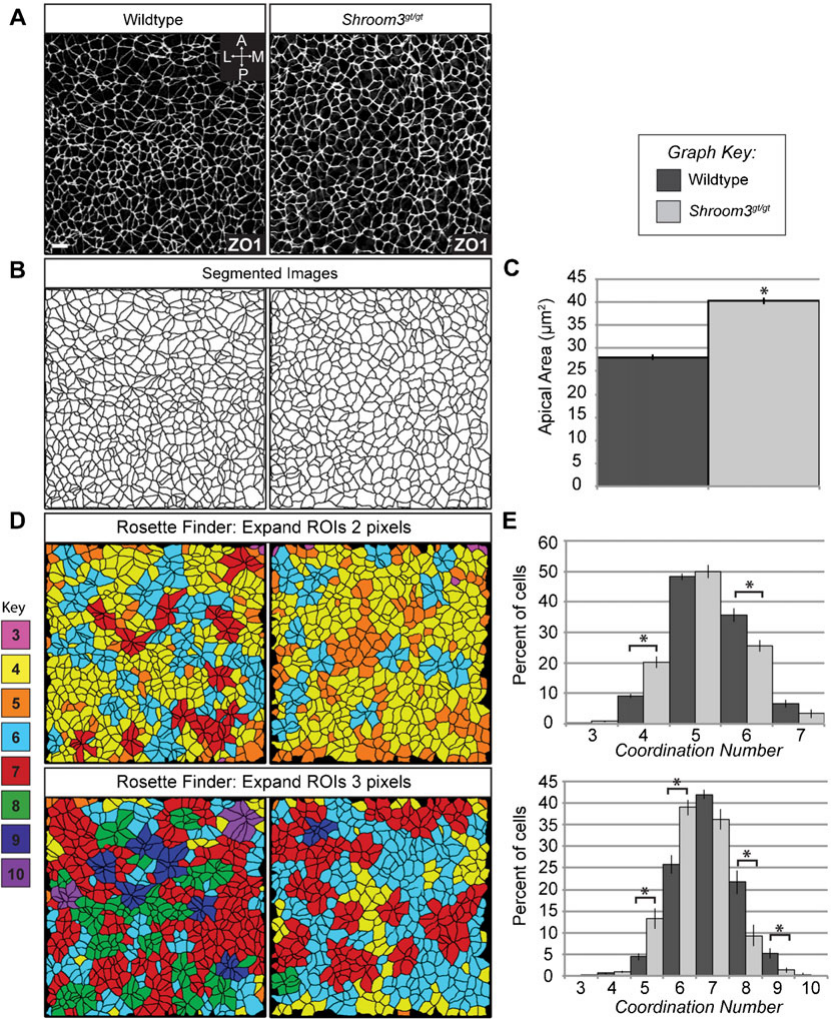
*Shroom3* mutants exhibit defective planar cell arrangement. (A,B) Wildtype (left panels) and *Shroom3^gt/gt^* (right panels) E8.5 embryos were stained to detect ZO1 (A) and the Seedwater Segmenter was used to segment confocal images. Scale bar: 10 µm. (B). Segmented images were imported into ImageJ and the segments converted into ROIs. (C) Graph shows average apical area of neural plate cells in 5 wildtype and *Shroom3^gt/gt^* embryos, n≥300 cells. (D) Rosette formation in wildtype (left panels) and Shroom3 deficient neural plates (right panels). Cells were color-coded based on the number of cells that meet at shared vertices (coordination number). (E) Cells were binned based on their coordination number and the percent of cells in each bin was plotted to compare the complexity of cell clusters. The overlap threshold was set at either 2 or 3 pixels and the results from both analyses are presented (top panels, 2 pixel overlap; bottom panels, 3 pixel overlap). *p<0.001.

Based on the above results, we hypothesized that Shroom3-induced tension at mediolateral junctions facilitates the formation of cellular rosettes to promote CE. To test this, we stained neural plates of E8.5 embryos to detect ZO1 to visualize the planar arrangement of cells. Images were then segmented [SeedWater Segmenter (Mashburn et al., 2012)] to produce separate Regions of Interest (ROI) for each cell (Fig. 10A,B). Analysis of segmented images indicates that *Shroom3* deficient cells exhibit a 48% increase in apical area (Fig. 10C), consistent with its role in apical constriction.

ROIs were further analyzed using a custom ImageJ macro to determine the number of cells that meet at shared vertices. This is referred to as the coordination number and represents the cellular complexity of the rosette, such that cells in rosettes will have a higher number of cells that share a common vertex. Coordination numbers were calculated based on cell overlap after the ROIs were expanded. Cell clusters were either color coded (Fig. 10D) or binned (Fig. 10E), based on their coordination number. Cells in multiple clusters were assigned the highest coordination number. In practice we found that changing the overlap threshold from 2 to 3 pixels increased the number of rosettes as well as the cellularity of each rosette. For comparison we present both analyses and find that they report qualitatively similar results. Both wildtype and *Shroom3* deficient neural plates exhibit clusters containing 3–10 cells, suggesting that Shroom3 is not required for rosettes (Fig. 10D). The percent of cells with each coordination number, relative to the total number of cells, was graphed and used to compare the complexity of rosettes between wildtype and *Shroom3^gt/gt^* embryos (Fig. 10E). This analysis demonstrates that *Shroom3* mutants exhibit a significant decrease in the complexity of the rosettes but not the total number of rosettes. Live-imaging studies of mouse neural plates and elongating kidney tubules have shown that loss of rosette complexity within the tissue results in a loss of cellular remodeling and neighbor exchange and an overall decrease in tissue extension (Lienkamp et al., 2012; Williams et al., 2014). Therefore, our data suggest that Shroom3 is likely required for the cellular remodeling that facilitates CE and neural tube closure.

## DISCUSSION

### Cooperation of the Shroom and PCP pathways

We have shown that the Shroom3 and PCP pathways interact to control the planar distribution of apical contractile actomyosin networks in epithelial cells of the neural plate. We predict that the asymmetric distribution of the Shroom3-Rock-Myosin II pathway facilitates the organization of cells into rosettes and the directional bending of the neural plate, both of which are implicated in neural tube closure. The data presented here suggest that the intersection of the Shroom3 and PCP pathways represents a model of how the simultaneous reduction of multiple pathways can cause NTDs. Based on our results, we hypothesize that the Shroom3 and PCP pathways work together during neural tube closure to define the subcellular distribution of activated, contractile actomyosin networks. In addition to the Shroom3 pathway, there appear to be additional mechanisms that ensure the robustness of neural tube closure. These include the Dvl2-PDZRhoGEF and the Ptk7-Rock pathways that have been shown to regulate Myosin II activity during neural tube closure (Nishimura et al., 2012; Andreeva et al., 2014). The presence of multiple pathways that all regulate Myosin II activity may account for the observations that neural tube closure defects in humans, while relatively common, are not typically linked to single mutations. It will be interesting to determine the genetic interactions that exist between these various pathways and explore how these networks may interact in the etiology of disease that impact human neural development.

### Physical interactions between the Shroom3 and PCP pathways

How PCP signaling is translated into changes in cell shape, cell topography, and contractility is an important area of research due to its relevance to a number of developmental processes associated with tissue morphogenesis. Studies of chick neural tube morphogenesis indicate that PCP signaling is linked to cellular organization and directional Myosin II activity by PDZ-RhoGEF (Nishimura et al., 2012). In this pathway, the formin DAAM1 recruits and activates PDZ-RhoGEF, resulting in the localized activation of RhoA along mediolateral junctions. This is predicted to activate Rock along these same junctions and trigger anisotropic actomyosin contractility that can be used to drive directional changes in cellular and tissue morphology. The work presented here provides evidence for a Dvl2-Shroom3-Rock pathway that also links planar polarization to directional contractility and cell organization during mouse neural tube closure. We have shown that Dvl2 can regulate the distribution of a Shroom3-Rock complex, providing a mechanism for PCP-mediated localization of Myosin II activity. This is supported by our observation that Dvl2 remains polarized in the absence of Shroom3 but that Shroom3 planar distribution is lost in a PCP mutant. Because Shroom3 is still localized to junctions, there must be other mechanisms involved in its localization, including direct interactions with F-actin at sites of cadherin-mediate cell-cell adhesion (Hildebrand, 2005; Plageman et al., 2011). There may also be a feedback mechanism in which Shroom3 could reinforce Dvl localization or junctional architecture. This prediction is based on our observations that Shroom3 deficient neural epithelia exhibit decreased amounts of junctional F-actin. This decrease in junctional F-actin could account for the reduced levels of junctional Dvl2 we observe in *Shroom3* mutants, since Dvl has been shown to co-localize with F-actin (Capelluto et al., 2002; Wiggan and Hamel, 2002). Finally, the actin-binding and bundling activity of Shroom3 could play a role in junctional F-actin architecture and this could be perturbed in *Shroom3* mutants. This concept of feedback regulation between Shroom, Rock, and F-actin is supported by experiments in *Drosophila* (Simões et al., 2014). Defining the role of F-actin in planar polarity and CE is further complicated by the observations that Cofilin proteins are essential for correct trafficking of PCP components (Mahaffey et al., 2013).

Our data further indicate that Shroom3 and Dvl2 may associate, either directly or indirectly, *in vivo* and that this requires the DEP domain of Dvl and a region of Shroom3 located between the SPR and SD1 domains. While a clear function for this region has not been identified previously, it has been shown to participate in signaling by the adaptor protein Posh during axon growth (Taylor et al., 2008). Interestingly, the corresponding region of Shroom2 has been shown to bind to ZO1 and Myosin VIIA (Etournay et al., 2007). Therefore, this region may represent a binding surface that links Shroom proteins to regulators or effectors and likely plays an important role in the spatial regulation of Shroom function.

### The role of Shroom3 in neural tube morphogenesis

Prior to this study, it was unclear if Shroom3 activity is regulated. We propose a model in which PCP signaling results in the accumulation of Dvl2, Shroom3 and Rock at mediolateral junctions in the neural plate to facilitate multiple aspects of neural tube closure. It should be pointed out that because Shroom3 is also localized to AP junctions, it may provide tension along both axes of the neural plate. First, at the cellular level, Shroom3 regulated actomyosin contractility could drive apical constriction. Consistent with this, we show that Shroom3 deficient neuroepithelial cells exhibit a significant increase in apical area at the time of neural tube morphogenesis. Anisotropic contraction could facilitate cell shortening along the mediolateral axis or participate in hinge point formation to change tissue shape. Second, we observe contiguous actomyosin cables oriented along the mediolateral axis. Contraction of these cables could promote either elevation or bending of the neural folds. Additionally, we predict that these contractile cables could provide tissue rigidity to withstand the external forces that participate in neural tube closure. Finally, contraction of mediolateral junctions could promote elongation of the body plan along the AP axis via the formation of higher order rosettes that facilitate directional cell rearrangements.

It is interesting to note that we still observe the formation of what appear to be cellular rosettes even in the absence of Shroom3 and its associated contractile networks. This is in contrast to what is observed in Drosophila embryos, where Shroom deficiency results in reduced rosette formation during germband extension (Simões et al., 2014). The fact that we do not observe a loss of rosette formation in the absence of Shroom3 is, however, consistent with recent studies indicating that rosette formation and resolution in mouse neural plate is not driven solely by apical contractility (Williams et al., 2014). However, data from this same report also suggests that apical Myosin IIb is not planar polarized in the neural plate. We would suggest that this discrepancy results from differences in what portion of the embryo was analyzed, as we focused on the cranial neural tube, while these data appear to be collected from the spinal region. Combined, these data suggest that the mouse neural tube and Drosophila ectoderm may have different requirements for apical contraction during rosette formation. It will be interesting to rectify these differences using additional mouse mutants that target these pathways pathway and live cell imaging to get a more complete picture of how rosette formation/resolution and apical contractility contribute to mouse neural tube morphogenesis.

## MATERIALS AND METHODS

### Mice and embryos

The *Shroom3* null allele (*Shroom3^gt(ROSA)53sor^*, *Shroom3^+/gt^*) has been described previously (Hildebrand and Soriano, 1999). LPT/LeJ (*Vangl2^+/Lp^*) and B6;129S7-*Wnt5^atm1Amc^*/J (Yamaguchi et al., 1999; Yamaguchi et al., 2011) mice were obtained from the Jackson Laboratory (Bar Harbor, ME, USA). PCR genotyping was performed using the following primers: Wnt5aF1: 5′-GAGGAGAAGCGCAGTCAATC-3′, Wnt5aF2: 5′-GCCAGAGGCCACTTGTGTAG-3′, Wnt5aR: 5′-CATCTCAACAAGGGCCTCAT-3′, Shroom3F: 5′-GGTGACTGAGGAGTAGAGTCC-3′, Shroom3R1: 5′-GAGTTTGTCCTCAACCGCGAGC-3′, Shroom3R2: 5′-GAGCACTGGCTGCTCTTCATG-3′, LP_F: 5′-GCTGGCCAAACAGTGGACCTTGGTTA-3′, LP_R: 5′-ACTGGCAGAAATGTGTCAGGGCCAG-3′. To genotype the *Lp* allele, PCR primers were designed to create a HpaI restriction endonuclease site including the *Lp* mutation (Neff et al., 1998). This line was genotyped by PCR followed by digestion and alleles were separated on 3% Metaphor agarose gel (Lonza, Rockland, ME, USA). Animal care and use was in accordance with the guidelines established by the University of Pittsburgh IACUC.

### Axis length measurements

Embryos from timed mating were dissected in PBS + 0.2% BSA, fixed in Bouin's Fixative for 2 hours, washed with 70% ethanol plus 0.1% NH_4_OH and photographed with an S6D dissecting scope equipped with a DFC 300F camera (Leica Microsystems, Wetzlar, Germany). Embryos were stained to visualize the fetal cartilaginous skeleton (Nagy et al., 2009) and photographed. Axis lengths were measured by drawing a line from the 2^nd^ cervical vertebrae to the pelvic girdle using ImageJ software (NIH, Bethesda, MD, USA) and taken as a ratio to the axis length of a wildtype littermate. The length of spina bifida was taken as a ratio to the overall axis length.

### Cell culture, transfection, and immunohistochemistry

COS7 fibroblasts were maintained in DMEM supplemented with 10% FBS, pen/strep, L-glutamine at 37°C and 5% CO_2_. Cells were transiently transfected with 1–2 µg of plasmid using Lipofectamine 2000 according to manufacturer's instructions (Invitrogen, Carlsbad, CA, USA). For immunohistochemistry, cells were plated on Fibronectin-coated cover slips at a density of 4×10^5^ cells and allowed to grow for 18–24 hours. Cells were washed with PBS, fixed with either −20°C MeOH or 4% PFA, and stained as previously described (Hildebrand, 2005). The following antibodies were used: rabbit anti-Shroom3 (1:200, UPT132, (Hildebrand, 2005), mouse anti-myc (1:200, 9E10, Developmental Studies Hybridoma Bank, Iowa City, IA, USA), mouse anti-Flag (1:200, Sigma, St Louis, MO, USA), and Alexa 488 or 568-conjugated secondary antibodies (1:400, Invitrogen). Cells were imaged using an Olympus Fluoview 1000 confocal microscope equipped with a 40× oil immersion objective (Olympus America, Center Valley, PA, USA). Two-dimensional projections were generated from *z*-series (0.5 µm steps) and images were processed using ImageJ.

### Molecular biology

pCS2 plasmids containing Shroom3L, Shroom3S, and myc-Shroom3SΔSD1 constructs have been previously described (Hildebrand and Soriano, 1999; Dietz et al., 2006). To generate Shroom3 deletion constructs, mShroom3 cDNA corresponding to amino acids 286–952, 286–776, 286–523, and 510–779 were cut from pCS2-Shroom3S plasmids and ligated into pCS3mt in frame with the myc tags. To generate GST-Shroom3 286–881, the cDNA of Shroom3 encoding amino acids 286–881 was PCR amplified and ligated into pGEX-3X.

### Co-immunoprecipitation and pull-down assays

Shroom3L (Hildebrand, 2005) and pCS2 flag-Dvl2 (Habas et al., 2001) expression plasmids were transiently transfected into COS7 cells and lysates were made in IP buffer (40 mM Tris pH 8.0, 5 mM EDTA pH 8.0, 10 mM EGTA pH 8.0, 100 mM NaCl, 0.5% Nonidet P-40, protease inhibitors). Lysates were precleared with anti-mouse agarose beads, incubated with mouse anti-flag antibody overnight at 4°C, followed by incubation with anti-mouse agarose beads for 4 hours at 4°C. The beads were washed in IP buffer, collected by centrifugation, resuspended in SDS sample buffer and resolved by SDS-PAGE. Proteins were transferred to a nitrocellulose membrane and detected using rabbit anti-Shroom3 (UPT132, 1:300) or mouse anti-flag (1:1000), HRP-conjugated secondary antibodies (1:2500, GE Healthcare Life Sciences, Pittsburgh, PA, USA), and ECL reagent (Thermo Fisher Scientific, Rockford, IL, USA). For GST-pull down experiments, pGEX-3X-Shroom3 286–881 plasmid was transformed into RIPL *E. coli* cells and protein expression was induced with 0.5 mM IPTG for 4 hours and purified as described (Farber et al., 2011). GST- Shroom3 286–881 bound to beads were incubated overnight with pre-cleared lysates made from COS7 cells transiently expressing flag-Dvl2. Beads were collected, washed in IP buffer, and resuspended in SDS sample buffer. Proteins were resolved by SDS-PAGE and analyzed via anti-Flag western blot.

### Whole mount immunohistochemistry of neural plates

E8.5 embryos were dissected in PBS + 0.1% FBS. For Shroom3 and Actin staining, embryos were fixed in 4% PFA in PBS overnight at 4°C and permeabilized in PBS + 0.5% Triton (PBT) for 30 min. For ZO1, Myosin IIb, Rock1, and Dvl2 staining, embryos were fixed in 80:20 Methanol:DMSO overnight, rehydrated in 50% MeOH for 30 min, washed in PBT (twice for 30 min), and blocked in PBS/5% BSA/10% heat-inactivated FBS for 1 hour. Embryos were incubated in primary antibody diluted 1:100 in block solution overnight. Antibodies used were rabbit anti-Shroom3 (UPT132; Hildebrand, 2005), rat anti-ZO1 (R26.4C, Developmental Studies Hybridoma Bank), rabbit anti-Myosin IIb (Covance, Princeton, NJ, USA) rabbit anti-Rock1 (Bethyl Labs, Montgomery, TX, USA), and rabbit anti-Dvl2 (Enzo Life Sciences, Farmingdale, NY, USA). Embryos were washed in PBT three times for 30 min each and incubated with secondary antibodies diluted in PBT overnight. Secondary antibodies used were Alexa 488 or 568-conjugated goat anti-rabbit or rat (1:400, Invitrogen). Tritc-phalloidin (1:500, Sigma) was used to detect actin. Embryos were washed with PBT and mounted in 2% agarose for imaging. Imaging was done using a Leica TCS SP5 confocal microscope equipped with an APO L 20×/1.00 water immersion objective. *Z*-series (1 µm steps) were collected and projections were generated using ImageJ.

### Analysis of planar distribution and cellular arrangement

Using ImageJ, 1-pixel wide user-drawn lines across cell junctions of non-saturated confocal projections were analyzed to determine the mean grey value and orientation relative to the mediolateral axis of the embryo. Those junctions between 0° and 45° or between 135° and 180° (Bins 1 and 4) were considered to be oriented along the mediolateral axis. Those between 45° and 135° (Bins 2 and 3) were considered to be oriented along the AP axis. Junctions were binned according to their angle and the fluorescence intensities in each bin were averaged. To quantify junctional versus medial F-actin fluorescence, the average fluorescence intensity was measured using either ROIs drawn along the junctions (junctional) or ROIs drawn inside the cell boundary (medial). For each experiment, at least 200 cells were analyzed from each of three embryos.

The semi-automated image-segmenting program Seedwater Segmenter (Mashburn et al., 2012) was used to segment images from five wildtype and five *Shroom3^gt/gt^* embryos to produce separate ROIs for each cell. Area measurements of each ROI were used to compare the average apical area of neural plate cells in wildtype versus *Shroom3^gt/gt^* embryos. n≥300 cells. ROIs with an average area of 800 pixels were expanded by 2 or 3 pixels and a custom image analysis tool was used to calculate the coordination number of each expanded ROIs. Cells were color-coded, binned based on their coordination number, and the percent of cells in each bin plotted.

### Statistical analyses

Measures of significance were determined by two-tailed, unpaired, Student's *t*-tests with p<0.001. For all graphs, error bars represent standard error of mean (±s.e.m.).
